# Visual Short-Term Memory Through the Lifespan: Preserved Benefits of Context and Metacognition

**DOI:** 10.1037/pag0000265

**Published:** 2018-08

**Authors:** Daniel J. Mitchell, Cam-CAN (Cambridge Centre for Ageing and Neuroscience), Rhodri Cusack

**Affiliations:** 1 Medical Research Council Cognition and Brain Sciences Unit, University of Cambridge; 3 University of Cambridge; 2 Brain and Mind Institute, Western University, and Trinity College Institute of Neuroscience, Trinity College, University of Dublin

**Keywords:** visual short-term memory, VSTM, aging, context, metacognition

## Abstract

Visual short-term memory (VSTM) ability falls throughout the life span in healthy adults. Using a continuous report task, in a large, population-based sample, we first confirmed that this decline affects the quality and quantity of reported memories as well as knowledge of which item went where. Visual and sensorimotor precision also worsened with advancing age, but this did not account for the reduced memory performance. We then considered two strategies that older individuals might be able to adopt, to offset these memory declines: the use of contextual encoding, and metacognitive monitoring of performance. Context and metacognitive awareness were both associated with significantly better performance, however these effects did not interact with age in our sample. This suggests that older adults retain their capacity to boost memory performance through attention to external context and monitoring of their performance. Strategies that focus on taking advantage of these preserved abilities may therefore help to maintain VSTM performance with advancing age. The article reports on analysis of the Cambridge Centre for Ageing and Neuroscience (Cam-CAN) data.

Visual short-term memory (VSTM), a critical part of everyday cognition, deteriorates from around 21 years of age, with item capacity halving by the age of 75 (Brockmole & Logie, 2013). As longevity increases, so does the importance of understanding and hopefully ameliorating this decline. When several items need to be held in mind over a few seconds, the number that can be remembered, and the precision with which each is remembered, both reduce through the adult life span (Noack, Lövdén, & Lindenberger, 2012; Peich, Husain, & Bays, 2013; Pertzov, Heider, Liang, & Husain, 2015). Furthermore, there are often increases in binding errors, in which the features (e.g., location and color) of two different objects are erroneously recombined (Cowan, Naveh-Benjamin, Kilb, & Saults, 2006; Mitchell, Johnson, Raye, Mather, & D’Esposito, 2000; Peich et al., 2013; but see Pertzov et al., 2015), perhaps reflecting a more general deficit in associative memory (Naveh-Benjamin, 2000). These age-related declines in VSTM performance have been found for various features (e.g., color/orientation/location) and encoding durations (e.g., 200 ms to 2,000 ms), although the age-related binding errors may be specific to reporting what went where, rather than bindings between nonspatial features (Brockmole, Parra, Della Sala, & Logie, 2008; Hoefeijzers, González Hernández, Magnolia Rios, & Parra, 2017; Parra, Abrahams, Logie, & Sala, 2009).

Given this reduction in the capacity, quality, and organization of VSTM, it might be that older adults do (or could) adopt compensatory strategies. In this article, we examine two potential sources of assistance. The first is context, which in younger adults has been found to improve VSTM performance. VSTM is often measured using change detection, by briefly presenting a sample display of items for memorization—which disappears for a maintenance period of around a second or more—followed by a probe display. If younger adults are asked to detect whether the color of a cued item changed between sample and probe, performance is better if it is presented in the probe display in the context of the items it appeared with during sample, rather than with no items, or with different items (Jiang, Olson, & Chun, 2000). This benefit from consistent context is also found in the broader memory literature, including verbal and episodic memory (Godden & Baddeley, 1975; Tulving & Thomson, 1973). The context effect in VSTM generalizes to memory for other features such as orientation, and across different types of spatial and nonspatial context (Hollingworth, 2006; Jiang, Chun, & Olson, 2004; Mutluturk & Boduroglu, 2014; Olson et al., 2004; Sligte, Scholte, & Lamme, 2008; Sun & Gordon, 2009). In a recent refinement (Rajsic & Wilson, 2014) memory was probed using a color report paradigm (Wilken & Ma, 2004), which allowed the precision as well as item capacity to be estimated (Bays, Catalao, & Husain, 2009; Zhang & Luck, 2008). It was found that the benefit of the context was to ensure the correct item was reported—in other words, to reduce color-location binding errors—rather than to affect the precision of color memory.

It is not known how the effect of context on VSTM changes with age. By providing information as to what went where, context could potentially confer an even greater benefit in older than younger adults, if it reduces binding errors that are otherwise more common in older adults. As a caveat, however, binding errors are not found in all circumstances and may depend on task details (Allen, Brown, & Niven, 2013; Brockmole et al., 2008; Hoefeijzers et al., 2017; Parra et al., 2009; Pertzov et al., 2015). Furthermore, it might be that older adults do (or could) more strongly exploit the strategy of elaborating on the relationships between items. For color memory, there is evidence that younger adults form multiple item (or ensemble) representations (Brady & Alvarez, 2011; Lin & Luck, 2009). In other types of working memory, older adults have been suggested to perform greater elaboration, as indexed by increased engagement of prefrontal brain regions during encoding (Reuter-Lorenz and Sylvester, 2005). This greater use of elaboration may lead to a greater benefit from context.

In other ways, context might confer less benefit to older adults. It has been shown that poorer attentional filtering affects memory performance in older adults, by leading to irrelevant items being processed more strongly and competing for memory (Gazzaley, Cooney, Rissman, & D’Esposito, 2005). If a context is presented at probe, the extra items might be encoded again and interfere with recall of the target item. Another mechanism through which context may be less beneficial for VSTM in older adults is if ensemble encoding is weakened by a reduction in associative memory (Naveh-Benjamin, 2000). Such a finding would be consistent with the discovery that in episodic memory, the encoding of context is weakened with age (Spencer & Raz, 1995). There is evidence that the benefit of spatial context for spatial VSTM is preserved in older adults (Olson et al., 2004), although this was for a memory measure that showed no age-related decline regardless of the context manipulation. Given these conflicting predictions, our first goal was to empirically investigate how the effect of context on VSTM changes with age.

The second factor that might be used to ameliorate age-related reductions in VSTM performance is metacognition—awareness of how well one is performing a mental task and the consequent regulation of strategy to improve performance (Borkowski, Carr, & Pressley, 1987; Flavell, 1979). In the context of memory this may be called *metamemory*. At least to some degree, young adults have accurate metacognitive awareness of VSTM representations and fluctuation in performance across trials (Adam & Vogel, 2017; Cowan et al., 2016; Fougnie, Suchow, & Alvarez, 2012; Rademaker, Tredway, & Tong, 2012; Suchow, Fougnie, & Alvarez, 2017; Vandenbroucke et al., 2014; but see Trübutschek et al., 2017 for a demonstration that nonconscious mechanisms can also support VSTM). In some situations, people are able to strategically reallocate resources across memory items (e.g., Atkinson, Baddeley, & Allen, 2017; Bays & Husain, 2008; Bengson & Luck, 2016; Fougnie, Cormiea, Kanabar, & Alvarez, 2016; Williams, Hong, Kang, Carlisle, & Woodman, 2013), and internal knowledge of the quality of memories can be used to guide such behavior (Suchow et al., 2017). Adults with better metacognition might therefore be able to adopt better strategies to adapt to changes in VSTM through the life span. For example, a strategy that may be helpful, and used especially by participants of higher fluid intelligence, is to attend to only a subset of items when presented with more than can be remembered (Atkinson et al., 2017; Cusack, Lehmann, Veldsman, & Mitchell, 2009; Linke, Vicente-Grabovetsky, Mitchell, & Cusack, 2011). Conversely, it may sometimes be beneficial to attend to the configuration of all items in the display, to the extent that these provide context, as described in the preceding text, that helps to anchor memories of individual items (Bengson & Luck, 2016).

Metacognitive abilities allow older adults to recruit compensatory strategies for episodic memory (Hertzog & Dunlosky, 2011). These could equally apply to VSTM, and may be particularly beneficial in older adults. However, metacognition itself may be affected by aging, and so might potentially be less beneficial in older adults (Nassar et al., 2016; Palmer, David, & Fleming, 2014). Our second goal, therefore, is to evaluate how metacognition for VSTM changes through the life span: whether good metacognition is predictive of better VSTM performance (i.e., do people with better metacognition tend to have better VSTM performance), and whether better metacognition predicts reduced changes in VSTM later in the life span.

Two further goals of the experimental design are noteworthy. First, a continuous color-report task was used to allow separation of the number of items that are remembered, the precision with which they can be remembered, and the probability of reporting the wrong item (Bays et al., 2009; Wilken & Ma, 2004; Zhang & Luck, 2008). Second, a well-matched perceptual/motor control was included, so that age-related changes in VSTM could be de-confounded from changes in color perception (e.g., due to changes in the eye), or motor control. As VSTM tends to decline from the 20s onward (Brockmole & Logie, 2013), we measured performance through the life span from 18 to 89, with approximately 100 participants per decade, to provide a reliable estimate throughout the range.

## Method

### Participants

A population-based sample of 700 healthy participants was recruited as part of the Cambridge Centre for Ageing and Neuroscience (Cam-CAN; see Shafto et al., 2014 for full details of the sample and exclusion criteria; all participants performed a range of psychological tests and neuroimaging assessments, but only the VSTM task is analyzed here). Of the participants recruited, 660 completed the VSTM task. Nine of these were excluded because of self-reported color-blindness; another 2 were excluded because of poor performance on the perceptual control condition (mean absolute error >30 degrees). The final sample thus consisted of 649 participants (337 female) with ages ranging from 18 to 89 (*M* = 54.7; approximately equal numbers per decile). The study was conducted in accordance with ethical approval obtained from the Cambridgeshire 2 (now East of England—Cambridge Central) Research Ethics Committee. Participants gave written informed consent.

### Stimuli and Task

**Figure 1 fig1:**
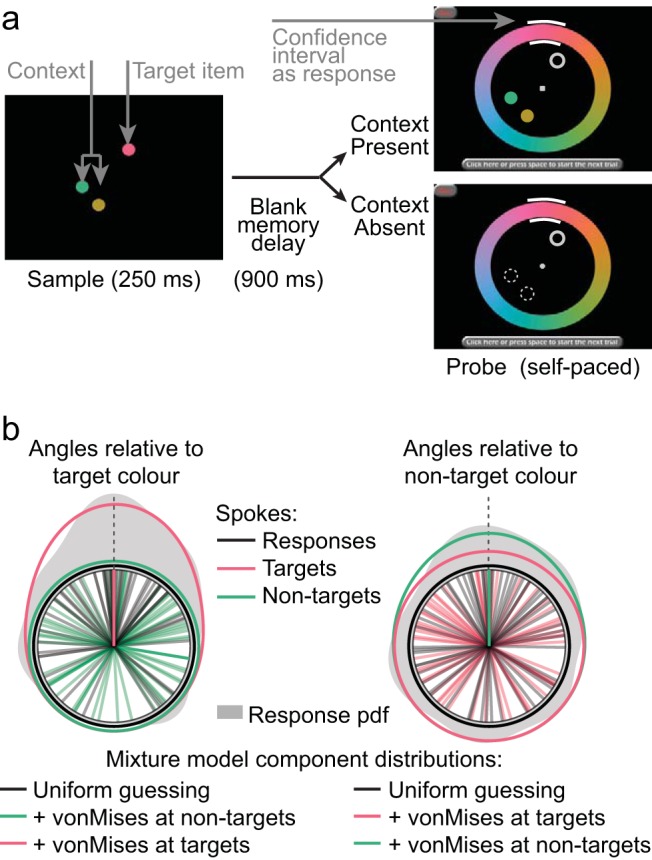
(a) Example trial, with memory load of three items, illustrated with and without context present at probe. (b) Illustration of mixture model, using the data from an example participant at a memory load of two items. On the left, angles are relative to the target color (pink spoke). Responses (gray spokes) are clustered around the target color, forming an error distribution (gray shading; probability density function, smoothed with a Gaussian kernel with standard deviation of 15 degrees). The error distribution is fit by a weighted mixture of a uniform distribution reflecting random guessing (black circle), plus von Mises distributions centered at nontarget colors (green circle), plus a von Mises distribution centered at target colors (pink distribution). The nontarget component is shown as a uniform distribution because nontarget colors are independent of (thus on average uniformly distributed with respect to) target colors. The right hand side replots the same data and mixture model fit, but with angles relative to the nontarget colors.

A continuous color report paradigm (Wilken & Ma, 2004; Zhang & Luck, 2008) was used, with an example trial illustrated in Figure 1a. Stimuli were generated using Visual Basic .NET and presented in a dimly lit room on a desktop PC running Windows XP, with a Higgstec 5-wire resistive touchscreen monitor.[Fig-anchor fig1]

On each trial, participants first saw a sample display, for 250 ms, which contained one to four colored disks on a black background. To ensure that similar distances around the color wheel on the screen corresponded to similar perceptual differences, colors used in the experiment were chosen from a circle in CIE L*a*b* color space, of radius 53 and center [64, 10, 10]. The diameter of each disk was approximately 1.77 degrees of visual angle (dva) and their positions were selected at random from eight equally spaced points at an eccentricity of 4.5 dva around a central, light gray, fixation cross. Following the brief sample display, a blank screen was presented for 900 ms, over which the sample items were to be held in memory.

A probe display then appeared, with the to-be-reported memory item cued by a thick gray outline at one of the disk locations. On half of trials, any uncued disks also reappeared, to provide the context within which the disk was encoded. On the remaining trials, other sample locations were marked by thin gray outlines, to provide disambiguating spatial information without color information. At the same time, a response color wheel was presented (radius 11 dva; thickness 4 dva). On each trial, the response wheel was randomly reflected and/or rotated by 0, 90, 180 or 270 degrees, which served two functions. First, it ensured that the memory could not be maintained as a motor memory for a specific preprepared response. Second, it avoided potential bias due to any consistent spatial shift on the touchscreen, from miscalibration or motor bias. Participants had as much time as required to select the color of the cued item, as accurately as possible, from the response wheel using a touchscreen. To capture metacognition about precision, we introduced a procedure whereby participants indicated their uncertainty in their choice of color by the length of time they touched the wheel: As they held their finger down, white confidence intervals spread out around the selected point indicating greater uncertainty about their selection. Participants were allowed to place multiple confidence intervals around the wheel if they could not decide between noncontiguous hues. Touching a button in the top left of the screen would delete the current selection(s) and allow participants to adjust their response, until they pressed the spacebar or a button at the bottom of the screen to proceed to the next trial. The fixation cross was then displayed during a 600-ms intertrial interval.

Before starting the main experimental blocks, participants completed a perceptual-motor control block of 56 trials with no memory requirement, in which single disks were presented at fixation along with the response wheel. The disk and response wheel both remained on screen until the participant reported the color of the disk by selecting a point on the response wheel. As soon as the participant touched the response wheel the next item appeared immediately. Trials with RTs of less than 300 ms were discarded. Accuracy on this task provided an estimate of individual differences in sensorimotor ability.

Following this control block, participants completed four practice trials of the VSTM task, with memory load increasing from one to four. Feedback was given in the form of the selected hue and the correct hue. Participants were allowed to repeat this practice if they wished, or if the experimenter felt that they did not understand the task. Performance on these practice trials was not analyzed further. Participants then completed two main blocks, of 112 VSTM trials each, in which no feedback regarding performance was given. Within the main VSTM blocks, memory load (1, 2, 3, or 4 items) and probe context were counterbalanced and randomly intermixed. The number of trials allowed the levels of these factors to be counterbalanced with the approximate color of the probed item: For each condition, one probe color came from each of 14 equally sized sectors uniformly distributed around the color wheel; within each sector, the precise hues were sampled at random. Any unprobed (nontarget) colors in each array were selected at random and independently of the probed color.

### Analysis

As a model-free index of performance, we calculated the response error—the angular difference between the target color presented and the color reported. This was summarized across trials as the root-mean-square error (RMSE). This model-free index cannot be used to distinguish errors due to imprecise memory of an item, from errors due to reporting the wrong item, or guessing when an item is not kept in memory at all. To estimate these, we also fitted a mixture model to the error distribution (see Figure 1b), to give measures of VSTM quantity (*K*, the expected number of items stored), quality (the precision of items held in memory) and misbinding (the probability of reporting a correct color from a wrong location). The mixture model we used was proposed by Zhang and Luck (2008), and modified by Bays and Husain (2008), and consisted of a component with a uniform distribution to account for random guesses, a von-Mises distribution (a circular analogue of the normal distribution) to describe the variability of responses to the target item and further von-Mises distributions centered at the hues of nonprobed items to account for nontarget responses (*misbinding*—reporting an item that was stored in memory but was not at the probed location). Maximum likelihood estimates of the mixture model parameters were obtained using code adapted from Bays and Husain (2008), using multiple starting points to avoid local minima. The concentration parameter of the von-Mises distribution was estimated using the method of Hassan, Hussin, and Zubairi (2012). We report the ‘precision’ of each item held in memory as the reciprocal of the standard deviation of the fitted von-Mises distribution. *K* is calculated by multiplying the memory load by the probability of responding from the target distribution. The measure of misbinding is given by the probability of responding from the nontarget distribution. These measures were derived separately per memory load and per participant, initially collapsing across context, and then separately for trials with and without probe context. The location parameter of the target von-Mises distribution was allowed to vary, but was set to zero for the nontarget distributions, so that the number of estimated parameters was constant across loads 2 through 4. Note that *K* can be interpreted empirically as the expected number of items stored, without taking a theoretical position on whether the underlying storage mechanism involves discrete “slots” or an infinitely divisible resource (Ma, Husain, & Bays, 2014).

For 3 participants at memory load 4, the probability of reporting the target item was estimated as zero. For these data points the precision is undefined so was excluded from the analysis. For each memory measure, any extreme outliers (more than six standard deviations from the mean) were also excluded (0 to 4 data points across measures). Although participants were allowed to make multiple guesses per trial, this was only done in 0.40% of trials across the entire dataset, so analyses used only the first response per trial.

The mixture model was also fit to errors on the control block to give estimates of sensorimotor ability in the absence of memory demands. Subsequent analyses were repeated with and without adjusting for individual differences in sensorimotor performance, by regressing both precision and *K* estimates from the control block out of each VSTM and confidence measure. (Having excluded people reporting color-blindness, nonzero probability of guessing in the control block is presumably due to accidently touching the screen in the wrong place, because no opportunity to adjust responses was given. Such “misclicks” are unlikely to be relevant in the VSTM task, in which people were able to correct and confirm their responses. Nevertheless, to be thorough in accounting for sensorimotor performance, both precision and *K* estimates from the control block were regressed out of the VSTM measures).

The benefit of consistent visual context between encoding and test displays was assessed using a linear mixed effects model, with a within-subject factor of probe context (present, absent), a between-subjects covariate of age, and memory performance as the dependent variable. Participants’ mean performance was modeled as a random effect to account for correlated errors and to generalize to the population. For each dependent memory measure, performance was summarized across memory loads, using the mean across loads for RMSE and precision, and the maximum across loads for *K* and misbinding, to avoid ceiling and floor effects at lower loads. An initial model fit used a linear age term. To capture potential nonlinear effects of age, quadratic age terms were added if warranted by a significant likelihood ratio test comparing the nested models (*p* < .05). This analysis was performed both with and without adjusting for sensorimotor effects of age. To adjust for sensorimotor decline, performance on the perceptual control task was regressed out of each memory measure, separately for each context condition.

To assess metacognitive awareness, the angular width of the reported confidence intervals provided a trial-by-trial measure of subjective uncertainty. To summarize overall uncertainty for each individual and condition, the mean was taken across trials. Participants with smaller values thus reported more confidence in their responses. Participants were not instructed that a particular width of their confidence intervals should correspond to a particular magnitude of estimated error, so it was not possible to determine metacognitive accuracy in an absolute sense. Rather, to capture the accuracy of each participant’s metacognitive awareness of fluctuations in memory performance across trials, we estimated the partial correlation between confidence and absolute actual error per trial, controlling for memory load and the presence or absence of context.

The benefit of metacognitive awareness was assessed using linear models, with memory performance as the dependent variable. Metacognitive awareness, age, and their interaction were entered as predictors. For each dependent memory measure, performance was summarized across memory loads as above. An initial model fit used a linear age term. To capture potential nonlinear effects of age, quadratic age terms were added if warranted by a significant *F* test comparing the nested models (*p* < .05). This analysis was performed both with and without adjusting for sensorimotor effects of age. Since age-related changes in sensorimotor performance could influence accuracy of confidence judgments as well as memory reports, performance on the perceptual control task was regressed out of the estimates of metacognitive awareness and each memory measure.

To quantify the relative evidence for the presence versus absence of main effects of context and metacognition, and their interactions with age, Bayes factors were calculated for each effect. The JZS Bayes factor was chosen (Rouder, Speckman, Sun, Morey, & Iverson, 2009), comparing the point null hypothesis to a nondirectional alternative hypothesis defined by a Cauchy prior distribution over standardized effect sizes. A value of 0.2 was chosen for the Cauchy scale parameter, such that the alternative hypothesis reflected an expectation that true effects had equal probabilities of being ‘small’ or being larger, according the definitions of Cohen (Cohen, 1988). In this way, a Bayes factor favoring a null result can be interpreted as no effect being x times more likely than a true effect (given equal prior probabilities), even when a true effect is likely to be small.

## Results

### All Measures of VSTM Performance Decline as Memory Load Increases and as Age Advances

**Figure 2 fig2:**
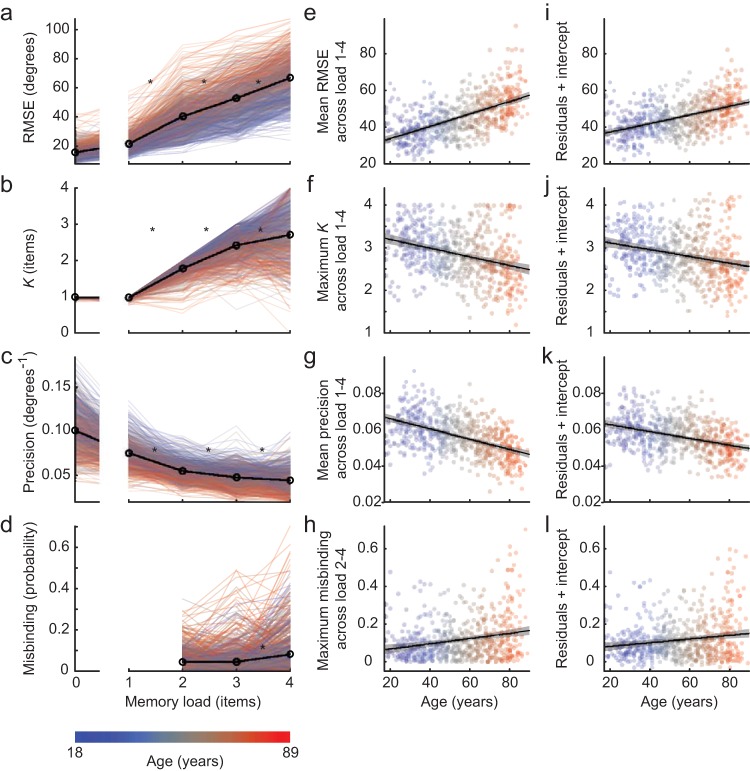
Effects of memory load and age on VSTM performance. (Panels a through d): Faint lines show performance of individual participants across memory loads, colored by age. The black line plots the mean across participants. Asterisks indicate significant changes between consecutive memory loads, *p* < .01, two-tailed, surviving Bonferroni correction across the three (Panels a through c) or two (Panel d) tests. (Panels e through h): Each measure is summarized across memory loads and plotted against age. The linear regression line is shown in black, along with its 99% confidence interval in gray. (panels i through l): As panels e through h, except that summary performance is first regressed against performance in the control task, and the residuals (plus intercept) are plotted against age.

As expected, greater memory load led to a worsening in performance (see Table 1), with an increase in raw error magnitude (Figure 2a). When partitioned into different aspects of VSTM performance, *K* increased on average with each increase in memory load, showing that more items were remembered as more were presented, but began to asymptote at higher loads as capacity limits were reached (see Figure 2b). The precision with which individual items were remembered also became worse with memory load, declining significantly with each increase in load, but also began to asymptote at higher loads (see Figure 2c). Finally, misbinding increased significantly from load 3 to load 4, but not from load 2 to load 3 (see Figure 2d).[Table-anchor tbl1][Fig-anchor fig2]

The effects of age are shown in Table 2 and Figure 2. Raw error magnitude significantly increased with age on average (see Figure 2e), and at all memory loads (see Figure 2a). *K* at each memory load reduced with age (see Figure 2b), but the relationship was weaker at lower loads due to a ceiling effect. To provide an estimate of each participant’s memory capacity for items, the maximum *K* across load was calculated (*K*_max_). This was dominated by high loads and thus not affected by the ceiling at lower loads. *K*_max_ significantly declined with age (Figure 2f). Items were also remembered less well, with precision significantly decreasing with age when averaged across loads (see Figure 2g) and at each memory load (see Figure 2c). Finally, misbinding was also summarized as the maximum across loads, a measure that was also dominated by higher loads thus reducing the floor effect at low loads. The maximum probability of misbinding significantly increased with age (see Figure 2h), as did misbinding probability at memory loads 2, 3 and 4 (see Figure 2d).[Table-anchor tbl2]

Some of the declining performance with age might be due to poorer color perception or motor control, rather than memory per se. To isolate effects specific to memory, we assessed performance on a control task that was matched in its sensory and motor demands, but had no memory component. This indeed showed a substantial age-related decline in the precision of responses, and a weaker though significant increase in the probability of random responses. To adjust for sensorimotor variability when assessing individual differences in VSTM performance, all correlations between age and VSTM were repeated after regressing both precision and *K* estimates from the control block out of every VSTM measure, and calculating the correlations using the residuals (see Table 2). Importantly, age-related declines remained for all memory measures (see Figures 2i through 2l).

### Context can Be Used to Boost All Measures of VSTM Performance, and the Benefit Is Preserved Across the Age Range

**Figure 3 fig3:**
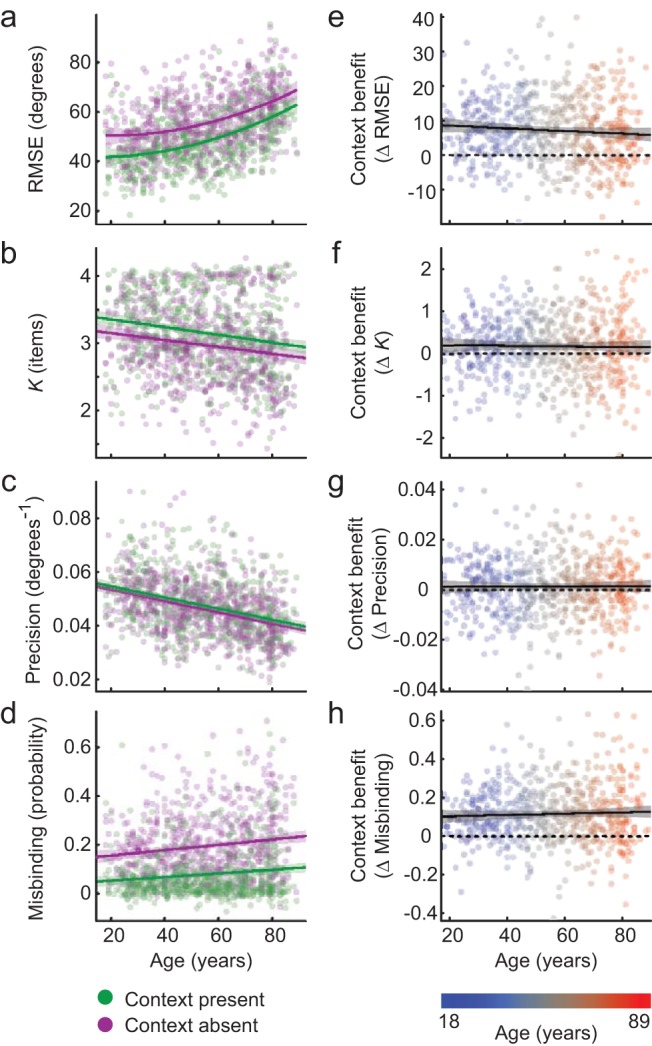
Effects of context on visual short-term memory (VSTM) performance, across the age range. Left (Panels a through d): Each measure is summarized across memory loads 2 through 4 then regressed against performance in the control task, and the residuals (plus intercept) are plotted against age. Performance with and without context reinstated at probe is shown in green and purple respectively. Lines show the fitted mean values for each context condition, along with their 99% confidence intervals. Right (Panels e through h): The per-participant context effect is plotted as the difference between context conditions, such that positive values correspond to a benefit from context. Linear regression lines are shown in black, along with 99% confidence intervals in gray.

Given the substantial changes in VSTM over the course of healthy aging, it is crucial to explore strategies that the aging population could use to offset these declines. First, we assessed the benefit from a visual context that matched across sample and probe displays. We assessed this using a linear mixed effects model with a within-subject factor of probe context (present, absent), a between-subjects covariate of age-at-test, and participant as a random effect (see Figure 3 and Table 3). A quadratic age term was added if it significantly improved the model fit. All four memory measures were summarized across memory loads 2 through 4 as described above, serving as the dependent variables. To adjust for potential sensorimotor effects of age, the analysis was repeated after regressing out performance on the perceptual control task, separately for each context condition.[Fig-anchor fig3][Table-anchor tbl3]

As expected, strong effects of age were present for all VSTM measures. For some memory measures, a nonlinear effect of age was observed, with the decline in performance accelerating with advancing age. An effect of context was also observed for all measures, with performance being better when the context matched between probe and sample. The effect size for context relative to that for age was greatest for the probability of misbinding and was minimal for precision. In terms of the number of reportable items, the presence of context increased *K*_max_ by 0.18 items on average, enough to compensate for approximately 30 years of age-related decline.

Crucially, there was minimal indication of the context benefit changing with age. Regardless of whether or not sensorimotor performance was adjusted for, the measures of *K*_*max*_, precision and misbinding showed no interaction of age and context, and Bayes factors indicated substantial evidence in favor of the null hypothesis. Results for the RMSE measure were less definitive. Without adjusting for sensorimotor performance, there was a weakly significant age-by-context interaction (*p* = .037), although the Bayes factor weakly favored the null hypothesis. Numerically, the context benefit on RMSE decreased slightly with age, although it only dropped by 33% over 71 years, from 8.7 to 5.8 degrees. Part of this reduction might reflect older adults being less able to derive a context benefit if they are less able to perceive the context precisely. Consistent with this possibility, after adjusting for sensorimotor performance, the age-by-context interaction became nonsignificant (*p* = .054); the Bayes factor remained equivocal, despite a numerical preference for the null hypothesis. Overall, the results suggest that older individuals remain able to make use of context to boost various aspects of VSTM performance, with no clear evidence of this ability changing substantially across the age range.

### Accurate Metacognitive Awareness of Variability in VSTM Performance is Associated With Better VSTM Performance, and This Association is Invariant Across the Age Range

**Figure 4 fig4:**
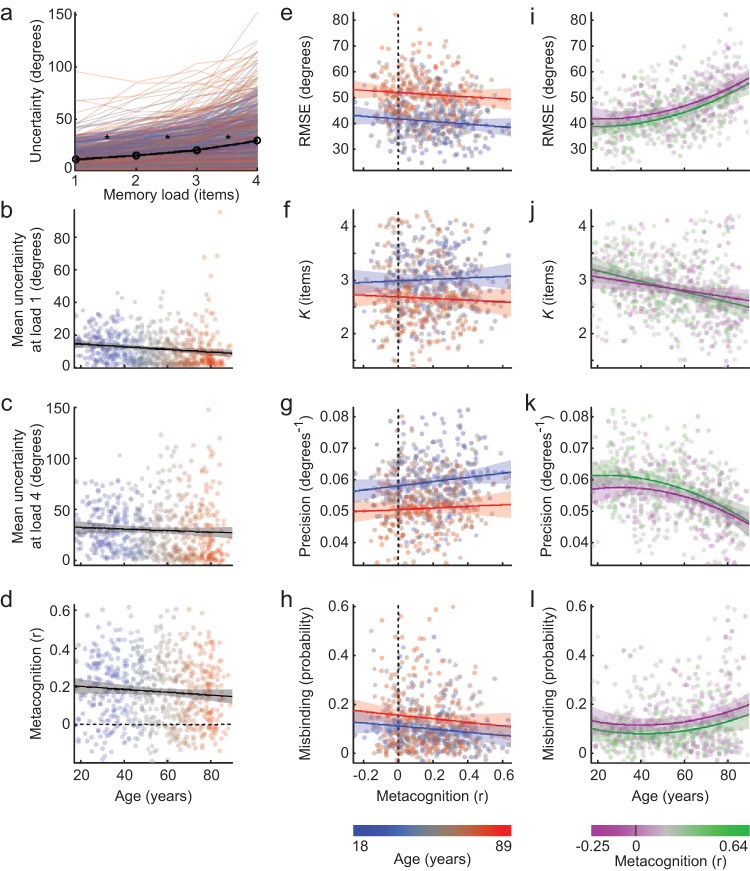
Analyses of uncertainty and metacognitive awareness across the age range. (Panel a) Faint lines show mean uncertainty for individual participants at each memory load, colored by age. The black line plots the mean across participants. Asterisks indicate significant increases between consecutive memory loads, *p* < .01, two-tailed, surviving Bonferroni correction across the three tests. (Panels b through d): Mean uncertainty at load 1 (Panel b), mean uncertainty at load 4 (Panel c), and metacognitive awareness of absolute error (Panel d) are plotted against age, after adjusting for individual differences in visual short-term memory (VSTM) performance (RMSE). Linear regression lines are shown in black, with 99% confidence intervals in gray. (Panels e through h): For each memory measure, summary performance across memory load is plotted against metacognitive awareness of absolute error, and colored by age. Blue and red lines, along with 99% confidence intervals, illustrate the relationship between performance and metacognition at low and high levels of age (blue: 15th percentile, age 33; red: 85th percentile, age 77). In all cases, memory performance and metacognitive awareness have been regressed against performance in the control task, and their residuals (plus intercept) are plotted. (Panels i through l): Same data as in Panels e through h, but replotted with age on the *x*-axis and colored by metacognitive awareness. Purple and green lines, along with 99% confidence intervals, illustrate the fitted relationship between performance and age at low and high levels of metacognitive awareness (purple: 5th percentile; green: 95th percentile).

We next turn to metacognition, the second factor that might modulate declines in VSTM. Mean ratings of subjective uncertainty were greater with increasing memory load (see Figure 4a), in line with increasing error magnitudes. Reported uncertainty declined slightly with age, but only at low memory loads (load 1: *r* = −0.14, *p* < .001; load 2: *r* = −0.13, *p* < .01; loads 3 through 4: *p* > .1). The same pattern held after adjusting for sensorimotor performance (load 1: *r* = −0.11, *p* < .01; load 2: *r* = −0.09, *p* < .05; loads 3 through 4: *p* > .1) or adjusting for individual differences in performance (RSME) on the VSTM task itself (see Figure 4b and 4c; load 1: *r* = −0.15, *p* < .001; load 2: *r* = −0.10, *p* < .01; load 3: *r* = −0.079, *p* < .05; load 4: *p* > .05). In other words, at lower loads, older adults are more confident of their judgments than younger adults, despite making larger errors. This *metacognitive bias* suggests that older adults may be unaware of their declining performance, consistent with reports of age-related increases in confidence, optimism and positivity more generally (Burns, Burns, & Ward, 2016; Chowdhury, Sharot, Wolfe, Düzel, & Dolan, 2014; Reed, Chan, & Mikels, 2014). It has also been suggested that a diminished capacity to represent uncertainty could lead to other age-related impairments in learning (Nassar et al., 2016).[Fig-anchor fig4]

Rather than just an overall sense of confidence, a more valuable type of metacognition in this task might be awareness of memory performance from trial-to-trial, which could allow people do dynamically adjust attentional strategies to optimize memory (Suchow et al., 2017). Such a measure also avoids a potential concern that individual differences in mean confidence might encompass differences in interpretation or implementation of the confidence judgment. For each participant, we therefore assessed metacognitive awareness of trial-wise memory performance by the relationship between objective and subjective accuracy across trials. This was quantified as the partial correlation between absolute error and subjective uncertainty, partialing out memory load and the presence of context.

Analyses were repeated with and without regressing out individual differences in sensorimotor performance from the measures of memory performance and metacognitive awareness. Without accounting for sensorimotor performance, estimates of age effects on metamemory might be inflated because, for example, people with poorer motor control would be less precise in recording both their memory and confidence judgments, even if the underlying judgments were accurate. On the other hand, adjusting for variance in sensorimotor performance might underestimate age effects on metamemory, because metacognition of sensorimotor performance may form an integral part of the overall metacogintive judgment, and because the adjustment would remove true memory-related variance across age that covaries with the sensorimotor differences. Regardless of adjusting for sensorimotor performance, adjusting for actual VSTM performance, or neither, metacognitive judgments were found to become less accurately predictive of performance with age (without adjusting for performance: *r* = −0.23, *p* < .001; after adjusting for sensorimotor performance: *r* = −0.14, *p* < .001; after adjusting for VSTM (RMSE) performance: *r* = −0.08, *p* < .05), but on average remained well above chance across the age range, and the decline was minimal after adjusting for VSTM performance (see Figure 4d).

Of greater interest, is how individual differences in metacognitive awareness relate to memory performance, and whether this relationship changes with age. We therefore used a linear model to predict memory performance from differences in metacognitive awareness, age, and their interaction. This was repeated for each memory measure, combining across memory loads as before, first without adjusting for sensorimotor ability and then with differences in sensorimotor performance regressed from both the memory measure and metacognitive awareness (see Figure 4d through 4g and Table 4). In addition to the expected effects of age, there was a significant negative association between metacognitive awareness of absolute error, and actual error (RMSE). That is, people who were better able to judge variability in their performance also tended to perform better. When compared to component memory measures, better metacognitive awareness was significantly associated with higher memory precision, and reduced probability of making misbinding errors, but was not associated with the maximum number of items that could be held in memory. Importantly, in no case was there a significant interaction between age and metacognitive awareness in predicting performance. Rather, Bayes factors indicated substantial evidence in favor of the null hypothesis that the association between metacognitive awareness and memory performance is not moderated by age. The same conclusions were reached whether or not data were adjusted for sensorimotor performance, although adjustment reduced the strength of the association between memory performance and metacognition.[Table-anchor tbl4]

The current measure of metacognitive awareness potentially combines quantitative knowledge of error magnitude with coarser knowledge of “remembered versus forgotten”, or whether items had been confused. To explore this, supplementary analyses measured metamemory as the correlation of uncertainty not with absolute error but with the trial-wise probabilities that the response came from the target distribution, nontarget distribution (misbinding) or uniform distribution (guessing). All measures of metamemory differed significantly from zero (all *t*[648] > 16.0, all *p*s < 3.0 × 10^−49^), suggesting that participants do have some awareness of these different types of error (although note that the trial-wise mixture probabilities are not independent of each other). Conclusions generally matched those for metacognition of absolute error (see Tables 1 and 2 and Figure 1 in the online supplemental material): Awareness of the probability of making a target response, or a random guess, were significantly associated with individual differences in RMSE and with the precision with which individual items were recalled, but not with *K*_*max*_; these associations did not change with age, and were robust to the adjustment for differences in sensorimotor performance. The only difference was that knowledge of trial-wise misbinding probability was not associated with individual differences in misbinding, and was only convincingly associated with individual differences in RMSE and precision before adjusting for sensorimotor performance. Therefore, although participants have accurate knowledge of whether they are guessing and whether they have forgotten which item went where, only the former is robustly associated with better memory performance. Overall, the results consistently suggest that people have some awareness of their VSTM performance across trials and, although this metacognitive awareness declines slightly with age, to the extent that it predicts better memory performance it does so similarly across the wide age range examined here.

## Discussion

In a large, representative sample of healthy adults across the life span, we characterized age-related decline in VSTM performance. Both the quantity of items in VSTM and the precision with which they were remembered declined with age, even after adjusting for reductions in sensorimotor accuracy. *Misbinding errors*—failing to recall what went where—also increased modestly with age. We then examined two compensatory strategies that older adults might be able to use to offset this decline. First, VSTM performance was enhanced when the context provided by the colors of the untested items was present in both the original sample and the probe display, with similar improvements observed across the age range. Second, people who had better metacognitive awareness of their trial-to-trial memory performance tended to perform better overall, with higher precision and fewer misbinding errors, and this relationship also persisted across the age range.

A recent study in young adults (Rajsic & Wilson, 2014) found that when consistent context was present across sample and probe then participants were more likely to report the correct item, but did not do so with higher precison. Here, we find that consistent context significantly improves all memory measures including precision. The difference in significance is likely due to the greater statistical power of the current study, and the current data are consistent with Rajsic and Wilson (2014) in that we find that the context effect on precision to be small in magnitude, compared to effects on the other memory measures.

Although the presence of consistent context enhances performance across the adult life span, this might at first glance appear to be of limited strategic use in the real world, where the supporting memoranda that are not the focus of recall may not easily be reinstated. Yet any focused task is embedded within a broad external context, and it may be sufficient to encode aspects of this broader context that are expected to remain constant during recall. Indeed, benefits derive from consistent context that is known to never itself be tested, both in in the case of VSTM (Hollingworth, 2006) and memory more broadly (Godden & Baddeley, 1975; Tulving & Thomson, 1973). In addition to leveraging preexisting external context, another strategy could be to add one’s own context to the items that need to be remembered, either physically, or by mental association. Use of such “internal context” may explain the VSTM advantage for recognisable objects, for which diverse contextual associations can more readily be formed (Veldsman, Mitchell, & Cusack, 2017). It would therefore be interesting to test whether the phenomenon of more precise recall of recognisable compared to unrecognisable objects might also be preserved throughout healthy aging. In the domain of long-term memory, the ‘Method of Loci’ is an ancient and powerful example of using mental spatial elaboration to enhance performance (Yates, 1966), although in this case the efficacy of the technique may decline with age (Verhaeghen & Marcoen, 1996).

Turning to metacognition, we found that while awareness of fluctuations in performance predicted individual differences in memory precision and misbinding errors, it was entirely unrelated to individual differences in memory capacity (*K*_max_). It is known that people do have awareness of the number of items that they can recall when they are explicitly asked to report this (Cowan et al., 2016; Rademaker et al., 2012). It is possible that the present task of quantifying uncertainty in recall of the tested hue may have focused introspection on precision, rather than the number of items in memory. In this case, an explicit judgment of the number of items stored may be found to correlate with individual differences in *K*_max._ Alternatively, precision and the fidelity of feature binding may be amenable to improvement via metacognitive strategies, whereas item capacity truly is not. It is also important to note that other aspects of metacognition are likely to be inportant in ageing. Although this experiment was not designed to measure absolute accuracy of metacognitive judgments, at low memory loads we observed a drop in overall uncertainty with age (despite, and regardless of, larger actual errors). This may reflect increasing overconfidence, which has been reported in other tasks and proposed to underlie learning deficits during healthy aging (Nassar et al., 2016).

Although context and metacognitive awareness were both associated with better VSTM performance, these effects did not interact with age in our sample (with the possible exception of a slight drop in context benefit on RMSE with age). Rather, Bayes factors always indicated evidence, typically substantial, in favor of the null hypotheses that contextual and metacognitive benefits to memory performance are not moderated by age. We anticipate that the age invariance of contextual and metacognitive benefits would ultimately break down beyond the age range examined here. Different relationships are likely to hold, for example, during childhood, when VSTM performance is improving (Burnett Heyes, Zokaei, van der Staaij, Bays, & Husain, 2012; Cowan et al., 2006; Gathercole, Pickering, Ambridge, & Wearing, 2004; Sarigiannidis, Crickmore, & Astle, 2016), and use of configural context has recently been shown to differ from adults (Cowan, Saults, & Clark, 2015). Performance is also expected to depend on different neural constraints during development and senescence (Sander, Lindenberger, & Werkle-Bergner, 2012). Nonetheless, the age invariance of contextual and metacognitive benefits appears to hold across an extremely wide age range, throughout the healthy adult life span.

This suggests that older adults might retain the potential to improve VSTM performance through attention to external context and by monitoring their performance. The experimental manipulation of context allows the inference that it causally boosts VSTM performance, however in the case of metacognition it is also possible that better memory facilitates more accurate metacognition, or that covariation between the measures is driven by a third factor. For example, the same process required to remember a color might be required to remember how good one’s memory about that color is. On the other hand, there is evidence that internal knowledge of memory quality can be used to redirect attention to specific memory items during the maintenance period, and that this can enhance recall of the prioritised item (Suchow et al., 2017). Any ability of metacognition to buffer the impact of age-related memory decline is likely to be even greater in situations where it could prompt the use of external memory aids as well as attentional strategies. These different causal hypotheses for the relation between individual differences in metacognition and VSTM performance cannot be distinguished by the current experiment, but are not mutually exclusive.

Conclusions were robust to whether or not individual differences in sensorimotor performance were regressed from the memory measures. If context and metacognitive ability can offset age-related decline in performance, then this is interesting whether it is achieved via cognitive or sensorimotor mechanisms. The residualized results provide a more conservative estimate of memory-related age differences aiming to focus on cognitive factors, but at the risk of removing true memory-related variance that is shared with sensorimotor variance across age. It is also important to bear in mind that although the adjustment accounts for measured sensorimotor performance it cannot perfectly control for the latent construct of sensorimotor ability (Westfall & Yarkoni, 2016).

Future work could investigate how the brain representations of visual memories change with age, and whether the effects of context and metacognitive strategies are to support brain representations that are more like those of younger adults, or whether they recruit distinct brain systems or representational mechanisms. A number of neural markers of individual differences in VSTM are available, using EEG (e.g., Sauseng et al., 2009; Vogel & Machizawa, 2004), MEG (e.g., Honkanen, Rouhinen, Wang, Palva, & Palva, 2015; Mitchell & Cusack, 2011; Palva, Monto, Kulashekhar, & Palva, 2010; Siebenhühner et al., 2016), and fMRI (e.g., Ester, Anderson, Serences, & Awh, 2013; Galeano Weber, Peters, Hahn, Bledowski, & Fiebach, 2016; Linke et al., 2011; McNab & Klingberg, 2008; Stevens, Tappon, Garg, & Fair, 2012; Todd & Marois, 2005; Veldsman et al., 2017; Vicente-Grabovetsky, Carlin, & Cusack, 2014), with some differences reported across age groups (for a review see Sander et al., 2012).

In a cross-sectional sample, as here, an observed change with age can include contributions from cohort effects, as well as physiological effects of aging. However, the benefits of context and metacognition on VSTM performance are found to be invariant to age, so it seems likely that they are invariant to both cohort effects and to longitudinal aging, unless these two factors had opposing effects that happened to cancel out. It would nevertheless be instructive to confirm this in a longitudinal sample. A longitudinal design could also help to disentangle the proportion of aging-related decline that is common to the memory and sensorimotor control tasks.

Although we report that the benefits of context and metacognition on VSTM performance are preserved across the adult life span, it remains to be demonstrated that older adults can proactively implement strategies to capitalize on these preserved abilities. For example, the ability of older adults to benefit from elaborative strategies in verbal associative memory is dependent on then having higher fluid intelligence (Frankenmolen et al., 2017). In the case of VSTM, performance can be boosted when strategy is guided by the simplest of instructions, in both students (Bengson & Luck, 2016) and older adults (Atkinson et al., 2017). We are therefore optimistic that strategic interventions, focused on attention to consistent context and self-monitoring of performance, may help to offset otherwise substantial declines in VSTM performance with advancing age.

## Supplementary Material

10.1037/pag0000265.supp

## Figures and Tables

**Table 1 tbl1:** Effect of Memory Load on VSTM Performance: Two-Tailed Paired t Tests Between Consecutive Memory Loads

Measure	Comparison	*t*(648)	*p*
RMSE	Loads 1 to 2	46.5	2.7 × 10^−208^
	Loads 2 to 3	29.2	3.1 × 10^−120^
	Loads 3 to 4	36.9	3.7 × 10^−161^
*K*	Loads 1 to 2	94.9	<1.0 × 10^−208^
	Loads 2 to 3	43.5	3.3 × 10^−194^
	Loads 3 to 4	11.4	9.3 × 10^−28^
Precision	Loads 1 to 2	34.7	1.2 × 10^−149^
	Loads 2 to 3	14.1	1.9 × 10^−39^
	Loads 3 to 4	5.53	4.6 × 10^−8^
Misbinding	Loads 2 to 3	.25	.804
	Loads 3 to 4	8.22	1.1 × 10^−15^
*Note*. VSTM = visual short-term memory; RMSE = root-mean-square error.			

**Table 2 tbl2:** Pearson Correlations of Age With VSTM Performance

Measure	Condition	Correlation with age		Correlation of residuals with age, after regressing performance on control task	
		*R*	*p*	*r*	*p*
RMSE	Mean across memory loads	.57	5.6 × 10^−58^	.47	4.1 × 10^−37^
	Control task	.34	2.7 × 10^−19^		
	Memory load 1	.40	7.8 × 10^−26^	.28	6.7 × 10^−13^
	Memory load 2	.46	7.1 × 10^−35^	.34	6.3 × 10^−19^
	Memory load 3	.51	3.2 × 10^−45^	.41	1.3 × 10^−27^
	Memory load 4	.56	2.9 × 10^−54^	.46	5.3 × 10^−36^
*K*	Max across memory loads	−.33	9.3 × 10^−18^	−.26	1.2 × 10^−11^
	Control task	−.12	2.0 × 10^−3^		
	Memory load 1	−.16	4.4 × 10^−5^	−.11	4.0 × 10^−3^
	Memory load 2	−.38	3.8 × 10^−19^	−.23	1.9 × 10^−9^
	Memory load 3	−.38	3.3 × 10^−23^	−.29	8.2 × 10^−14^
	Memory load 4	−.34	2.1 × 10^−19^	−.27	1.1 × 10^−12^
Precision	Mean across memory loads	−.49	2.7 × 10^−41^	−.38	8.3 × 10^−24^
	Control task	−.31	1.2 × 10^−15^		
	Memory load 1	−.37	8.0 × 10^−23^	−.24	5.3 × 10^−10^
	Memory load 2	−.39	6.2 × 10^−25^	−.28	2.8 × 10^−13^
	Memory load 3	−.37	3.1 × 10^−22^	−.28	3.8 × 10^−13^
	Memory load 4	−.32	2.0 × 10^−16^	−.25	1.7 × 10^−10^
Misbinding	Max across memory loads	.23	3.1 × 10^−9^	.17	1.7 × 10^−5^
	Memory load 2	.22	2.3 × 10^−8^	.14	4.0 × 10^−3^
	Memory load 3	.18	5.6 × 10^−6^	.12	1.7 × 10^−3^
	Memory load 4	.14	5.6 × 10^−4^	.11	7.2 × 10^−3^
*Note*. VSTM = visual short-term memory; RMSE = root-mean-square error.					

**Table 3 tbl3:** Effects of Context, Age, and Their Interaction on VSTM Performance

		Without adjusting for sensorimotor performance				After adjusting for sensorimotor performance			
VSTM measure	Effect	Coefficient	*F*(1,1293–1,1294)	*p*	Bayes factor	Coefficient	*F*(1,1293–1,1294)	*p*	Bayes factor
RMSE	Intercept	50.7				51.5			
	Age	.408	344	2.4 × 10^−68^	3.4 × 10^64^ favoring H_1_	.289	195	2.2 × 10^−41^	7.2 × 10^37^ favoring H_1_
	Age^2	6.30 × 10^−3^	24.3	9.5 × 10^−7^	1.3 × 10^4^ favoring H_1_	3.79 × 10^−3^	9.96	1.6 × 10^−3^	13 favoring H_1_
	Context	−3.63	414	5.0 × 10^−80^	1.4 × 10^76^ favoring H_1_	−3.63	414	4.4 × 10^−80^	1.6 × 10^76^ favoring H_1_
	Age:Context	.0205	4.38	.037	1.1 favoring H_0_	.0189	3.73	.054	1.5 favoring H_0_
*K*	Intercept	3.07				3.07			
	Age	−6.88 × 10^−3^	56.6	1.0 × 10^−13^	5.7 × 10^10^ favoring H_1_	−5.43 × 10^−3^	35.3	3.7 × 10^−9^	2.4 × 10^6^ favoring H_1_
	Context	.0911	40.8	2.4 × 10^−10^	3.3 × 10^7^ favoring H_1_	.0911	40.8	2.3 × 10^−10^	3.4 × 10^7^ favoring H_1_
	Age:Context	−5.56 × 10^−4^	.504	.48	7.2 favoring H_0_	−2.96 × 10^−4^	.146	.70	8.6 favoring H_0_
Precision	Intercept	.0481				.0469			
	Age	−2.97 × 10^−4^	258	4.9 × 10^−53^	2.4 × 10^49^ favoring H_1_	−2.08 × 10^−4^	139	1.8 × 10^−30^	1.3 × 10^27^ favoring H_1_
	Age^2	−3.73 × 10^−6^	12.0	5.4 × 10^−4^	36 favoring H_1_				
	Context	6.72 × 10^−4^	9.06	2.7 × 10^−3^	8.6 favoring H_1_	6.72 × 10^−4^	9.11	2.6 × 10^−3^	8.8 favoring H_1_
	Age:Context	−6.02 × 10^−6^	.243	.622	8.2 favoring H_0_	1.94 × 10^−6^	.0253	.87	9.1 favoring H_0_
Misbinding	Intercept	.126				.137			
	Age	1.39 × 10^−3^	44.9	3.1 × 10^−11^	2.36 × 10^8^ favoring H_1_	9.22 × 10^−4^	20.4	6.9 × 10^−6^	2.0 × 10^3^ favoring H_1_
	Age^2	3.06 × 10^−5^	6.43	.011	2.4 favoring H_1_				
	Context	−.0578	408	4.3 × 10^−79^	1.6 × 10^75^ favoring H_1_	−.0578	409	3.3 × 10^−79^	2.1 × 10^75^ favoring H_1_
	Age:Context	1.65 × 10^−4^	1.11	.29	5.4 favoring H_0_	−1.74 × 10^−4^	1.23	.27	5.1 favoring H_0_
*Note*. Results of linear mixed effects model, with the age variable centered and levels of context coded as [1, −1]. VSTM = visual short-term memory; RMSE = root-mean-square error.									

**Table 4 tbl4:** Effects of Metacognitive Awareness (of Absolute Error), Age, and Their Interaction on VSTM Performance

		Without adjusting for sensorimotor performance				After adjusting for sensorimotor performance			
VSTM measure	Effect	Coefficient	*F*(1,644–1,645)	*p*	Bayes factor	Coefficient	*F*(1,644–1,645)	*p*	Bayes factor
RMSE	Intercept	43.7				44.4			
	Age	.326	285	3.6 × 10^−53^	2.8 × 10^49^ favoring H_1_	.234	175	1.6 × 10^−35^	1.1 × 10^32^ favoring H_1_
	Age^2	5.22 × 10^−3^	23.0	2.0 × 10^−6^	7.0 × 10^3^ favoring H_1_	3.43 × 10^−3^	11.6	7.0 × 10^−4^	35 favoring H_1_
	Metacognition	−8.48	18.7	1.7 × 10^−5^	954 favoring H_1_	−4.65	6.27	.013	2.8 favoring H_1_
	Age:Metacog.	−.101	.880	.35	4.4 favoring H_0_	.0251	.0580	.81	6.4 favoring H_0_
*K*	Intercept	2.84				2.84			
	Age	−.0100	71.5	1.9 × 10^−16^	2.4 × 10^13^ favoring H_1_	−7.96 × 10^−3^	46.7	1.9 × 10^−11^	3.7 × 10^8^ favoring H_1_
	Metacognition	.0754	.382	.54	5.5 favoring H_0_	−7.79 × 10^−3^	8.53 × 10^−4^	.98	6.6 favoring H_0_
	Age:Metacog.	−4.2 × 10^−3^	.400	.53	5.5 favoring H_0_	−6.67 × 10^−3^	.914	.34	4.3 favoring H_0_
Precision	Intercept	.0574				.0568			
	Age	−2.70 × 10^−4^	181	1.5 × 10^−36^	1.1 × 10^33^ favoring H_1_	−1.87 × 10^−4^	103	1.8 × 10^−22^	1.6 × 10^19^ favoring H_1_
	Age^2	−4.97 × 10^−6^	19.8	1.0 × 10^−5^	1.6 × 10^3^ favoring H_1_	−3.33 × 10^−6^	10.3	1.4 × 10^−3^	19 favoring H_1_
	Metacognition	8.66 × 10^−3^	18.8	1.7 × 10^−5^	965 favoring H_1_	4.67 × 10^−3^	6.04	.014	2.6 favoring H_1_
	Age:Metacog.	−5.05 × 10^−5^	.208	.65	6.0 favoring H_0_	−9.55 × 10^−5^	.786	.38	4.6 favoring H_0_
Misbinding	Intercept	.103				.106			
	Age	1.28 × 10^−3^	26.7	3.2 × 10^−7^	3.8 × 10^4^ favoring H_1_	9.52 × 10^−4^	15.5	3.4 × 10^−5^	209 favoring H_1_
	Age^2	4.12 × 10^−5^	9.38	2.3 × 10^−3^	12 favoring H_1_	3.38 × 10^−5^	6.53	.011	3.2 favoring H_1_
	Metacognition	−.0765	10.0	1.6 × 10^−3^	16 favoring H_1_	−.0688	7.78	5.4 × 10^−3^	5.8 favoring H_1_
	Age:Metacog.	−5.60 × 10^−4^	.176	.67	6.1 favoring H_0_	−2.10 × 10^−4^	.233	.88	6.5 favoring H_0_
*Note*. Result of linear model, with the metacognitive awareness and age variables centered. VSTM = visual short-term memory. RMSE = root-mean-square error.
