# Supplementary material for: Visual Short-Term Memory Through the Lifespan: Preserved Benefits of Context and Metacognition
Source: Psychol Aging. 2018 Aug;33(5):841–54. doi: 10.1037/pag0000265 (PMC6084281; doi:10.1037/pag0000265)
Supplement: Supplementary file 1 [file PAG-2017-1351Supp.zip › cc700_vstm_v13_revision_PA_160318_supp.docx]

| Awareness of probability of… | | …Target response | | | …Guessing from uniform distribution | | | …Non-target response | | |
| --- | --- | --- | --- | --- | --- | --- | --- | --- | --- | --- |
| VSTM Measure | Effect | Coefficient | p | Bayes factor | Coefficient | p | Bayes factor | Coefficient | p | Bayes factor |
| RMSE | Intercept | 43.7 |  |  | 43.6 |  |  | 43.7 |  |  |
|  | Age | 0.328 | 4.6x10^-54^ | 2.2x10^50^ for H_1_ | 0.333 | 1.1x10^-55^ | 8.6x10^51^ for H_1_ | 0.335 | 3.1x10^-56^ | 3.0x10^52^ for H_1_ |
|  | Age^2 | 5.41x10^-3^ | 7.5x10^-7^ | 1.7x10^4^ for H_1_ | 5.54x10^-3^ | 4.4x10^-7^ | 2.8x10^4^ for H_1_ | 5.44x10^-3^ | 6.7x10^-7^ | 1.9x10^4^ for H_1_ |
|  | Metacognition | 9.66 | 4.4x10^-6^ | 3.4x10^3^ for H_1_ | -8.05 | 1.1x10^-4^ | 175 for H_1_ | -10.1 | 2.9x10^-4^ | 76 for H_1_ |
|  | Age:Metacognition | 0.0640 | 0.58 | 5.7 for H_0_ | -0.0784 | 0.50 | 5.3 for H_0_ | 0.0223 | 0.90 | 6.6 for H_0_ |
| *K* | Intercept | 2.84 |  |  | 2.84 |  |  | 2.84 |  |  |
|  | Age | -0.0102 | 3.5x10^-17^ | 1.2x10^14^ for H_1_ | -0.0101 | 6.1x10^-17^ | 7.0x10^13^ for H_1_ | -0.0104 | 1.3x10^-17^ | 3.1x10^14^ for H_1_ |
|  | Metacognition | 0.0264 | 0.86 | 6.5 for H_0_ | 0.0558 | 0.65 | 6.0 for H_0_ | -0.0990 | 0.66 | 6.0 for H_0_ |
|  | Age:Metacognition | 6.19x10^-3^ | 0.39 | 4.7 for H_0_ | -2.83x10^-3^ | 0.69 | 6.2 for H_0_ | -0.0152 | 0.15 | 2.5 for H_0_ |
| Precision | Intercept | 0.0574 |  |  | 0.0574 |  |  | 0.0574 |  |  |
|  | Age | -2.69x10^-4^ | 4.7x10^-37^ | 3.3x10^33^ for H_1_ | -2.74x10^-4^ | 1.6x10^-38^ | 9.5x10^34^ for H_1_ | -2.79x10^-4^ | 2.7x10^-39^ | 5.5x10^35^ for H_1_ |
|  | Age^2 | -5.14x10^-6^ | 4.2x10^-6^ | 3.6x10^3^ for H_1_ | -5.23x10^-6^ | 3.0x10^-6^ | 4.8x10^3^ for H_1_ | -5.06x10^-6^ | 6.5x10^-6^ | 2.4x10^3^ for H_1_ |
|  | Metacognition | -0.0110 | 2.6x10^-7^ | 4.7x10^4^ for H_1_ | 8.97x10^-3^ | 1.8x10^-5^ | 936x10^#^ for H_1_ | 9.82x10^-3^ | 4.2x10^-4^ | 54 for H_1_ |
|  | Age:Metacognition | 1.08x10^-4^ | 0.37 | 4.5 for H_0_ | -6.53x10^-5^ | 0.58 | 5.7 for H_0_ | -1.80x10^-3^ | 0.30 | 4.0 for H_0_ |
| Misbinding | Intercept | 0.103 |  |  | 0.102 |  |  | 0.102 |  |  |
|  | Age | 1.33x10^-3^ | 1.0x10^-7^ | 1.1x10^5^ for H_1_ | 1.33x10^-3^ | 7.0x10^-8^ | 1.4x10^5^ for H_1_ | 1.45x10^-3^ | 6.1x10^-9^ | 1.6x10^6^ for H_1_ |
|  | Age^2 | 4.45x10^-5^ | 9.8x10^-4^ | 26 for H_1_ | 4.50x10^-5^ | 8.6x10^-4^ | 29 for H_1_ | 4.58x10^-5^ | 7.0x10^-4^ | 34 for H_1_ |
|  | Metacognition | 0.0663 | 0.010 | 3.4 for H_1_ | -0.0780 | 2.0x10^-3^ | 14 for H_1_ | -7.91x10^-3^ | 0.80 | 6.4 for H_0_ |
|  | Age:Metacognition | -4.58x10^-4^ | 0.75 | 6.3 for H_0_ | 2.46x10^-4^ | 0.86 | 6.5 for H_0_ | 2.09x10^-3^ | 0.81 | 6.4 for H_0_ |

*Supplementary Table 1.* Effects of metacognitive awareness (of trial-wise mixture model probabilities), age, and their interaction, on VSTM performance. Results of linear model, with the metacognitive awareness and age variables centred. Measures of metacognitive awareness and VSTM performance are *not* adjusted for sensorimotor performance.

| Awareness of probability of… | | …Target response | | | …Guessing from uniform distribution | | | …Non-target response | | |
| --- | --- | --- | --- | --- | --- | --- | --- | --- | --- | --- |
| VSTM Measure | Effect | Coefficient | p | Bayes factor | Coefficient | p | Bayes factor | Coefficient | p | Bayes factor |
| RMSE | Intercept | 44.4 |  |  | 44.4 |  |  | 44.4 |  |  |
|  | Age | 0.234 | 1.1x10^-35^ | 1.4x10^32^ for H_1_ | 0.235 | 4.2x10^-36^ | 3.8x10^32^ for H_1_ | 0.238 | 1.2x10^-36^ | 1.3x10^33^ for H_1_ |
|  | Age^2 | 3.47x10^-3^ | 5.6x10^-4^ | 42 for H_1_ | 3.50x10^-3^ | 5.2x10^-4^ | 45 for H_1_ | 3.43x10^-3^ | 6.6x10^-4^ | 36 for H_1_ |
|  | Metacognition | 5.99 | 2.5x10^-3^ | 11 for H_1_ | -5.10 | 8.5x10^-3^ | 3.9 for H_1_ | -5.58 | 0.029 | 1.4 for H_1_ |
|  | Age:Metacognition. | -0.0255 | 0.82 | 6.5 for H_0_ | -9.57x10^-3^ | 0.93 | 6.6 for H_0_ | 0.116 | 0.46 | 5.2 for H_0_ |
| *K* | Intercept | 2.84 |  |  | 2.84 |  |  | 2.84 |  |  |
|  | Age | -8.04x10^-3^ | 9.4x10^-12^ | 7.2x10^8^ for H_1_ | -7.90x10^-3^ | 1.8x10^-11^ | 3.9x10^8^ for H_1_ | -8.14x10^-3^ | 7.6x10^-12^ | 8.9x10^8^ for H_1_ |
|  | Metacognition | 0.108 | 0.44 | 5.0 for H_0_ | -3.64x10^-4^ | 0.97 | 6.6 for H_0_ | -0.213 | 0.29 | 3.9 for H_0_ |
|  | Age:Metacognition. | 8.04x10^-3^ | 0.28 | 3.8 for H_0_ | -3.97x10^-3^ | 0.59 | 5.8 for H_0_ | -0.0170 | 0.11 | 2.0 for H_0_ |
| Precision | Intercept | 0.0568 |  |  | 0.0568 |  |  | 0.0568 |  |  |
|  | Age | -1.86x10^-4^ | 2.3x10^-22^ | 1.3x10^19^ for H_1_ | -1.86x10^-4^ | 9.5x10^-23^ | 3.1x10^19^ for H_1_ | -1.91x10^-4^ | 2.3x10^-22^ | 1.2x10^20^ for H_1_ |
|  | Age^2 | -3.38x10^-6^ | 1.1x10^-3^ | 23 for H_1_ | -3.41x10^-6^ | 1.0x10^-3^ | 25 for H_1_ | -3.29x10^-6^ | 1.6x10^-3^ | 17 for H_1_ |
|  | Metacognition | -7.25x10^-3^ | 3.3x10^-4^ | 67 for H_1_ | 6.07x10^-3^ | 1.9x10^-3^ | 14 for H_1_ | 5.05x10^-3^ | 0.051 | 1.1 for H_0_ |
|  | Age:Metacognition | 1.25x10^-4^ | 0.28 | 3.8 for H_0_ | -7.71x10^-5^ | 0.50 | 5.3 for H_0_ | 1.78x10^-4^ | 0.28 | 3.8 for H_0_ |
| Misbinding | Intercept | 0.105 |  |  | 0.105 |  |  | 0.105 |  |  |
|  | Age | 9.84x10^-4^ | 5.8x10^-5^ | 320 for H_1_ | 9.75x10^-4^ | 6.1x10^-5^ | 308 for H_1_ | 1.05x10^-3^ | 1.7x10^-5^ | 960 for H_1_ |
|  | Age^2 | 3.60x10^-5^ | 6.7x10^-3^ | 4.8 for H_1_ | 3.63x10^-5^ | 6.2x10^-3^ | 5.2 for H_1_ | 3.64x10^-5^ | 6.3x10^-3^ | 5.1 for H_1_ |
|  | Metacognition | 0.0575 | 0.027 | 1.5 for H_1_ | -0.0704 | 5.2x10^-3^ | 6.0 for H_1_ | 3.38x10^-3^ | 0.94 | 6.6 for H_0_ |
|  | Age:Metacognition. | -7.37x10^-4^ | 0.62 | 5.9 for H_0_ | 5.03x10^-4^ | 0.73 | 6.3 for H_0_ | 5.63x10^-4^ | 0.79 | 6.4 for H_0_ |

*Supplementary Table 2*. Effects of metacognitive awareness (of trial-wise mixture model probabilities), age, and their interaction, on VSTM performance. Results of linear model, with the metacognitive awareness and age variables centred. Measures of metacognitive awareness and VSTM performance *are* adjusted for sensorimotor performance.


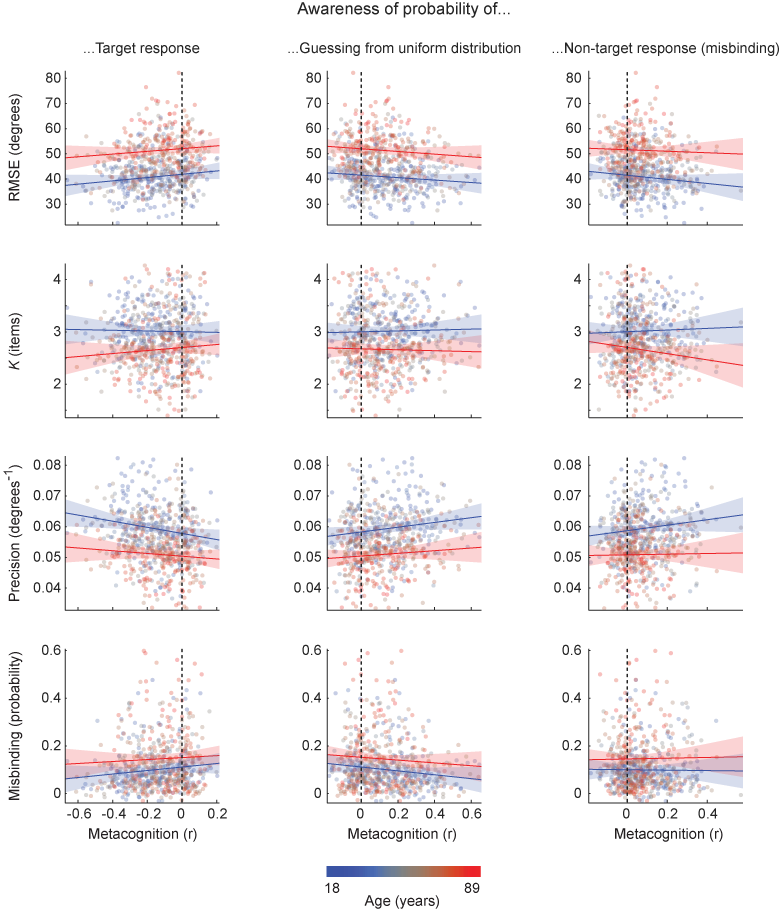
 *Supplementary Figure 1. Effects of metacognitive awareness (of trial-wise mixture model probabilities), age, and their interaction, on VSTM performance. Each panel shows a different combination of measures of objective memory performance (rows) and subjective awareness of error probabilities (columns). In each panel, summary performance across memory load is plotted against metacognitive awareness, and coloured by age. Blue and red lines, along with 99% confidence intervals, illustrate the fitted relationship between performance and metacognition at low and high levels of age (blue: 15th percentile, age 33; red: 85th percentile, age 77). In all cases, memory performance and metacognitive awareness have been regressed against performance in the control task, and their residuals (plus intercept) are plotted.*
